# Global One Health post-graduate programmes: a review

**DOI:** 10.1186/s42522-024-00097-6

**Published:** 2024-04-10

**Authors:** Olayide Abraham Adeyemi, Tariq Oluwakunmi Agbabiaka, Hasnat Sujon

**Affiliations:** 1 Faculty of Veterinary Medicine, University of Ilorin, Ilorin, Nigeria; 2 Microbiology Unit, Department of Science Laboratory Technology, Federal Polytechnic, Damaturu, Nigeria; 3 Directorate General of Health Services, Ministry of Health and Family Welfare, Dhaka, Bangladesh

**Keywords:** One Health, Curricula, Master programme, Core competencies

## Abstract

**Background:**

The One Health (OH) approach recognises that humans, animals, plants, and the environment are interrelated, and therefore seeks to facilitate collaboration, communication, coordination, and capacity building between relevant stakeholders to achieve a healthier ecosystem. This calls for integrating OH into established governance, policy, health, education, and community structures, and requires OH professionals equipped with the necessary inter and trans-disciplinary skillset. Therefore, numerous OH training programmes are currently being offered globally. However, the coordination and contents of some of these trainings have been criticised as inconsistent and inadequately standardised, and therefore could serve as a barrier to OH implementation. In this study, an up-to-date repository of a subset of OH academic programmes offered globally was provided, and their curricula contents was critically assessed.

**Methods:**

Between December 2022 and April 2023, an online search for key terms ‘ONE HEALTH MASTERS COURSES’, and ‘ONE HEALTH MASTERS PROGRAMMES’ together with variations of ‘AFRICA’, ‘NORTH AMERICA’, ‘ASIA’, ‘AUSTRALIA’, ‘EUROPE’, 'GLOBAL' was conducted. Details about course title, delivery mode, joint administration status, curricula contents, language of instruction, years to completion, host university, country, and continent were collected.

**Results:**

Forty-three programmes met inclusion criteria of the study, and almost all (*n* = 36, 83.7%) were tailored towards infectious diseases and population/global health, compared to the environmental and conservation perspectives. Compiled curricula contents clustered into one of these 12 sub-headings: ‘principles and concepts of OH’, ‘epidemiology and biostatistics’, ‘major branches of OH’, ‘internship/externship/research project’, ‘infectious diseases, zoonoses, and surveillance’, ‘risk analysis and crises management’, ‘food safety, microbiology, immunology, and allied’, ‘communication’, ‘ethics’, ‘economics, policy, and management’ and ‘others. Of these, infectious disease themes were the most common. Regarding geography and organising institutions, North America and Europe, and veterinary institutions, respectively, were the most represented.

**Conclusion:**

Despite the multi-level diversity observed, uniformity still exists across the programmes which favours interdisciplinary cross-talks. Future pedagogical studies that objectively assess the alignment of module contents with the OH core competencies and the impacts of these OH programmes is recommended. With this study, a critical information gap that has existed for long in the OH field has been bridged.

**Supplementary Information:**

The online version contains supplementary material available at 10.1186/s42522-024-00097-6.

## Background

Over the past few decades, the concept of One Health (OH) approach has steadily risen both in prominence and acceptance, with a significant re-surge from 2010 onwards [1]. Currently, governments and policymakers are embracing this approach to consolidate lessons learnt from the COVID-19 pandemic and find sustainable solutions to numerous emerging health threats [2]. This widespread adaptation of the principles of OH, however, has led to growing concerns on how best to operationalise this concept for optimal public health security, ecosystem balance, amongst others [1, 3].

Ever since the late twentieth century, when the OH concept was introduced into public discourse, until recently, arriving at a unified OH definition proved elusive amongst the prior stakeholders of the concept [2, 4, 5]. More recently, the One Health High Level Expert Panel (OHHLEP), initiated by the formerly tripartite (now quadripartite) group, addressed this concern with their more balanced definition that has so far been widely adopted across disciplines. They defined OH as “an integrated, unifying approach to sustainably balance and optimise the health of humans, animals, plants, and ecosystems…through (the mobilisation of) multiple sectors, disciplines, and communities…to foster well-being and tackle threats to health and ecosystems, while addressing the collective need for healthy food, water, energy, and air, taking action on climate change and contributing to sustainable development” [6]. It can thus be taken as a consensus that OH aims to facilitate communication, collaboration, capacity building and coordination between relevant stakeholders towards achieving and sustaining improved health of people, animals, plants and entire ecosystems.

**Fig. 1:**
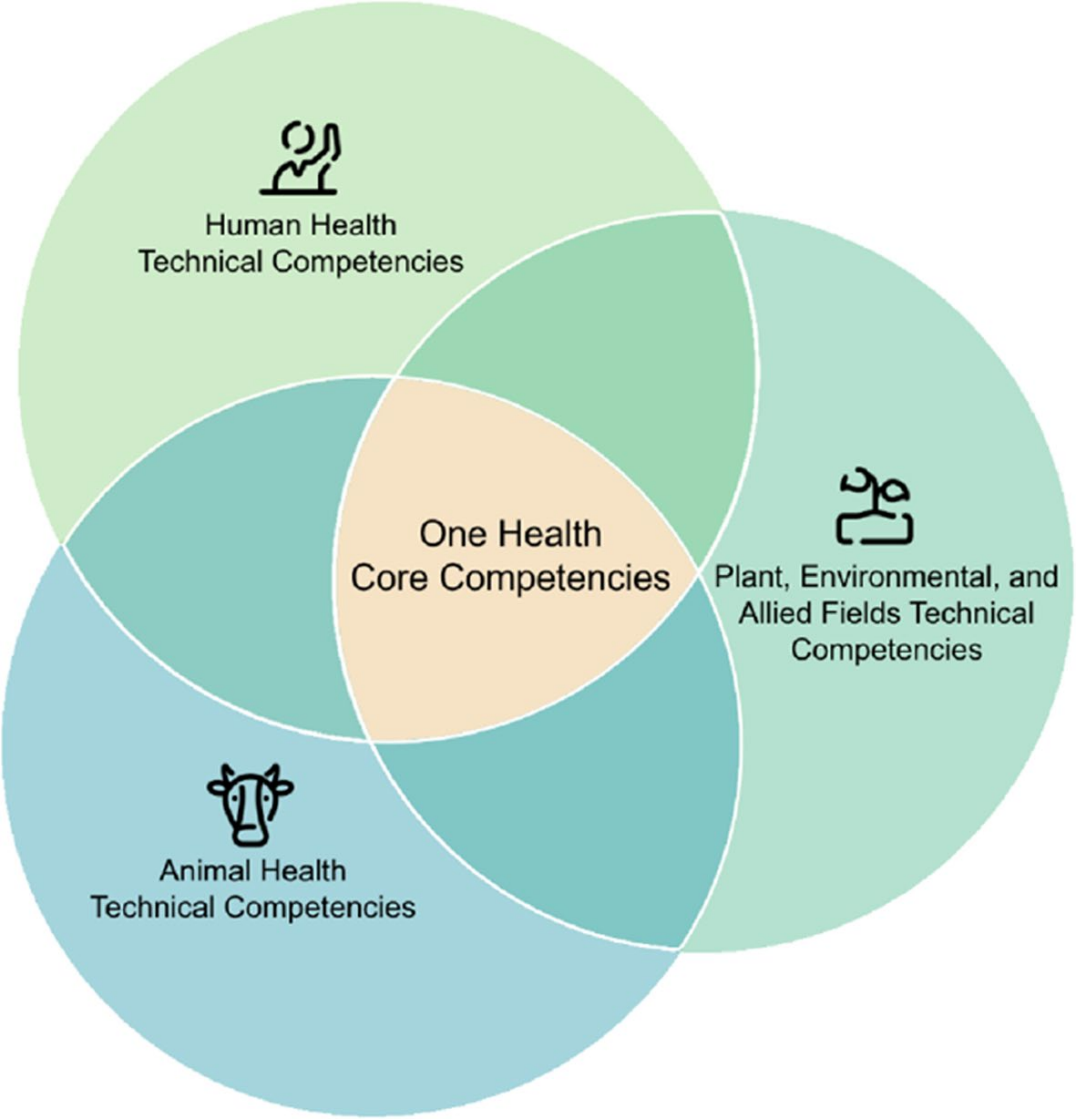
One Health (OH) core competencies. These are possessed by OH professionals from all the disciplines to ensure efficient cross-talk and collaboration between and across different disciplines involved [7]. Although there is a new OH stream of thought as suggested in Laing, et al., [10], this evolving concept still acknowledges that OH core competencies are common to all OH stakeholders as opposed to their unique professional competencies. This commonality is what the Venn diagram describes. The authors deliberately designed the diagram so that no individual component would be regarded as the major component

To achieve this aim, the OH approach needs to be integrated into established governance, policy, health, education, and community structures. The success of this integration is dependent on OH professionals equipped with the necessary inter and trans-disciplinary skill forte [4]. To this end, one of the major achievements made by OH pioneers of the past decades is the itemisation of such skills. Various independent OH stakeholders converged in what is now described as the ‘2012 Rome synthesis’ and concluded on seven ‘core competencies’, which entails an outline of skills and behaviours required of an OH practitioner [7]. These competencies include management, communication and informatics, values and ethics, leadership, teams and collaboration, roles and responsibilities, and systems thinking. Essentially, OH practitioners must also be equipped with these ‘core competencies’ in addition to competencies specific to their different disciplines [7] (Fig. 1). Recently, some OH stakeholders (including Amuguni et al., [8], Togami et al., [9] have highlighted gaps in the ‘Rome synthesis’ core competencies and have likewise recommended improvements. In response to such suggestions, the Network for Ecohealth and One Health (NEOH) recently proposed an update of the previous seven ‘core competencies’ into nine, which include effective communication,collaborative and resilient working,systems understanding,transdisciplinarity,social, cultural and gender equity and inclusiveness,collective learning and reflective practice,One Health concepts,theoretical and methodological pluralism,and harnessing uncertainty, paradox and limited knowledge. Like the prior ‘core competencies’, the NEOH’s hope is that this new update would guide OH curricula contents and professional training [10].

With this obvious need for adequately skilled OH professionals, numerous OH training programmes are being organised and administered globally to bridge this gap. These OH trainings are delivered formally as bachelor's or graduate degrees, diplomas, specialisation tracks, or through other less formal means including professional continuous education for public health practitioners and policymakers [11]. Similarly, non-profit initiatives such as One Health Lessons (website) [12] and One Health for One Planet Education (1HOPE) (website) [13] are contributing to the decentralization of OH education through their respective activities. However, the organisation of these OH programmes and the content of various OH trainings have become the subject of scrutiny and criticism. As earlier indicated, the OH approach seeks to foster cross-talks across various fields, and this makes it important for OH professionals to have a certain common understanding despite their varying backgrounds. In contrast to this expectation, Togami et al., [9] observed inconsistencies like the underrepresentation of important disciplines and a curriculum with a narrower focus in some OH training in the United States. Furthermore, the quadripartite group also noted that limited standardisation of OH curricula, other OH educational frameworks, and the ensuing OH workforce, could serve as a barrier to the implementation of OH although details about these limitations were not specifically indicated [14].

Presently, limited literature exists on the structure and contents of OH training programmes around the world, and only a minority of these studies have objectively analysed the structure of curricula contents and also studied their alignments with the OH core competencies [8, 9]. It is upon this sparse data, that OH educators and stakeholders (including the quadripartite group) are now left to draw objective conclusions about the extent of disparity in various OH training. In this decade, the OH field is quickly morphing, as stakeholders grapple with the practicalities of what OH professionals are, and what skills they should possess. On one hand, this fast-changing pace could be said to have contributed (in part) to the currently limited studies on the subject, while on the other hand, it should also necessitate the need for regular ‘time point’ analyses.

In this study, an up-to-date repository of a subset of OH academic programmes offered globally is provided. Curricula contents were summarised and critically reviewed to identify the uniformity and diversity within these programmes. The OH focus areas and professional backgrounds that are well or less represented in OH academic programmes were also highlighted. Lastly, these findings were employed to speculate on the career trajectories of students participating in the programmes and recommendations were made on how to balance the diversity of these programmes with the uniformity necessary to ensure that the global OH workforce produced possess the required minimal common understanding of OH essential to work jointly. As such, this study is confined only to the analysis of curricula content, with curricula mapping and in-depth pedagogical analysis falling outside the research scope.

## Methodology

Before the review of the different OH postgraduate courses that are organised and offered in higher academic institutions globally, a set of inclusion criteria for the courses to be analysed were first defined. OH courses that (1) clearly state ‘One Health’ in their title, or in the degree title to be awarded upon completion, and (2) that are delivered as either a master or a joint bachelor and master programme were primarily included. In addition, other courses that clearly stated in their synopsis how OH was integrated into their curricula were also included, and most programmes in this latter category are regular master in public health courses with a OH track option. An exception to include three Bachelor of Science OH programmes was also made. This eventual subset of OH courses considered in this study was well structured, and consequently allowed for proximate, thorough, and systematic analysis of curricula contents. However, these inclusion criteria is limited because of the exclusion of other OH training in the form of modules, summer schools, diplomas, and those outside the traditional educational framework. It is also important to note that although various stakeholders have recognised overlaps in the objectives and systems approach of EcoHealth and Planetary Health in relation to OH, each of these are still considered as distinct disciplines [15]. Due this technicality, the analysis was limited to the above-stated criteria.

Between December 2022 and April 2023, a thorough search for OH courses on Google and Microsoft Bing search engines was conducted. The key terms searched were ‘ONE HEALTH MASTERS COURSES’, and ‘ONE HEALTH MASTERS PROGRAMMES’ together with variations of ‘AFRICA’, ‘NORTH AMERICA’, ‘ASIA’, ‘AUSTRALIA’, ‘EUROPE’, ‘GLOBAL’. For each search syntax permutation, the suggested pages were followed until the hits became irrelevant.

Courses that fit the inclusion criteria and their corresponding weblinks were collated into Microsoft Excel and after a preliminary assessment, repeated programmes (as in the case of jointly organised programmes) were merged, and OH programmes that have been discontinued (as of April 2023) were further excluded. For the programmes with scanty or non-existent curriculum details in the public domain, templated emails were sent to the programme coordinators to solicit these details, but in cases where the coordinators were not reachable, such programmes were excluded.

After a final list of courses that fully met the inclusion criteria were collated, further details about each course, including course title, mode of delivery, joint administration status, curricula contents, language of instruction, years to completion, host university, country, and continent were collected. Lastly, the curricula contents (i.e. modules) across all programmes were organised into different clusters based on their commonalities, and also using the technical core skills classification of Amuguni et al., [8] as a reference point.

## Results

**Fig. 2:**
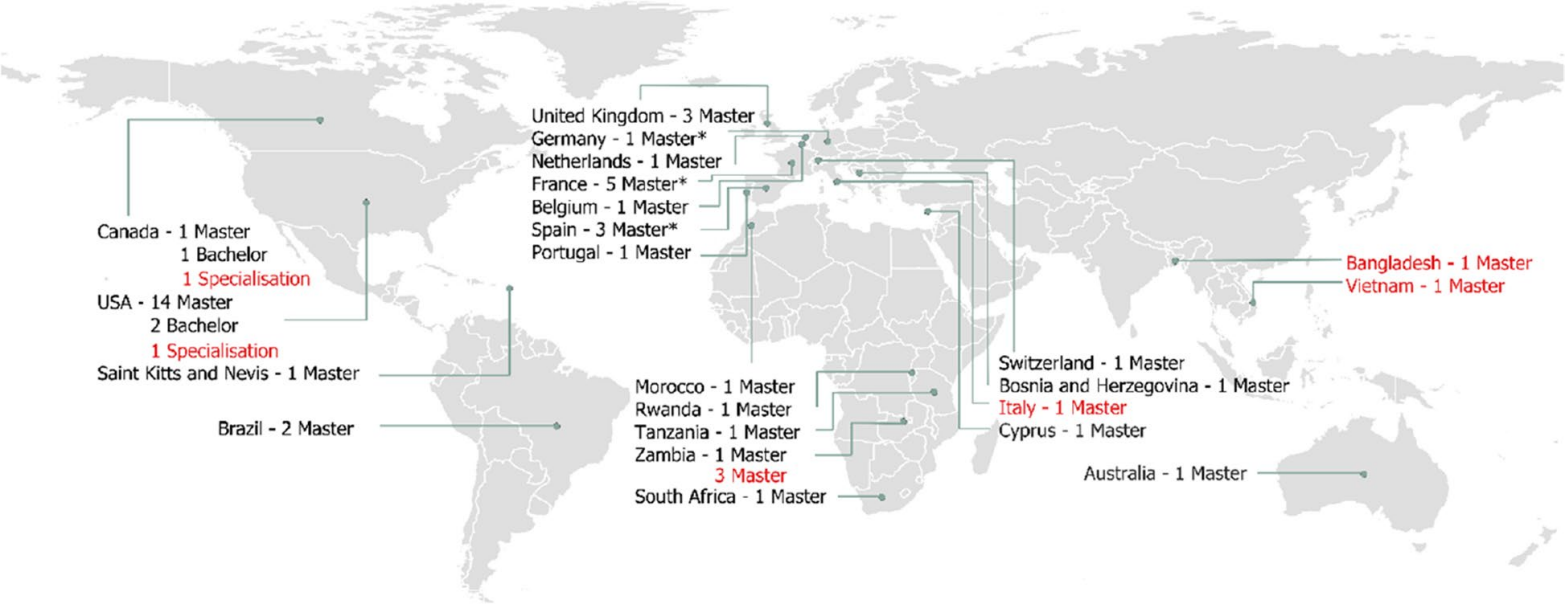
Geographical distribution of all 43 One Health (OH) programmes analysed in the current study. Most programmes were offered in North America (*n* = 19), followed by Europe (*n* = 16), Africa (*n* = 5), South America (*n* = 2) and Australia (*n* = 1). Except for three programmes organised in North America, all programmes analysed are master's degrees, or joint bachelor's and master's degrees. *One master programme is jointly offered by France, Spain, and Germany. Courses in black ink are included in the analysis; courses in red ink are not included in the analysis due to the unavailability of course curriculum and/or were not a master or bachelor programme, i.e., Specialisation courses (traineeships/short courses)

A total of 43 programmes met the inclusion criteria and were included in this study. Although these programmes were globally distributed, the majority are concentrated in North America and Europe (*n* = 19, 44.2% and *n* = 16, 37.2% respectively), and a minority are offered in Africa (*n* = 5, 11.6%), South America (*n* = 2, 4.7%) and Australia (*n* = 1, 2.3%). There are countries with multiple academic institutions offering OH programmes, and most of these countries with the highest representation here are from the global north, including the United States (*n* = 16, 37.2%), followed by France (*n* = 5, 11.6%), and each of the UK and Spain (*n* = 3, 7.0%) (Fig. 2).

Two-thirds of the programmes (*n* = 27, 63.0%) require a mandatory onsite presence of students, 23.3% (*n* = 10) are organised wholly virtual, and 11.6% (*n* = 5) provided a hybrid option of either online or onsite attendance. Also, about half of the programmes (*n* = 22, 51.2%) were available just to full-time participants, roughly one-third (*n* = 15, 34.9%) offer a blended option between full-time and part-time while one-eight minority (*n* = 5, 11.6%) offer only a part-time option.

English is the predominant language of instruction, and most programmes that are organised in non-English speaking countries, also offer English options to international participants. Less than 8.0% (*n* = 3) are delivered solely in other national languages (French and Portuguese). In addition, 14.0% (*n* = 6) of the courses analysed were jointly organised by at least two partner institutions (which might either be academic, research, or public). The most common time to completion is between one and two years (*n* = 35, 81%) except the bachelor programmes with a standard four-year timeframe, and other part-time options.

All the compiled curricula contents (i.e. modules) fitted into one of 12 clusters, and each was assigned the most representative sub-heading as follows: (1) principles and concepts of One Health, (2) epidemiology and biostatistics, (3) major branches of One Health, (4) internship/externship/research project, (5) infectious diseases, zoonoses, and surveillance, (6) risk analysis and crises management, (7) food safety, (8) microbiology, immunology, and allied, (9) communication, (10) ethics, (11) economics, policy, and management and (12) others (see Supplementary table 1). Here, the ‘Principles and concepts of OH’ was based on the OHHLEP definition earlier described, and the ‘Major branches of OH’ was defined as ‘ecosystem, human and animal health’ components – taught in the context of OH. However, considerable overlaps exists between at least two or more of these sub-headings.

**Fig. 3:**
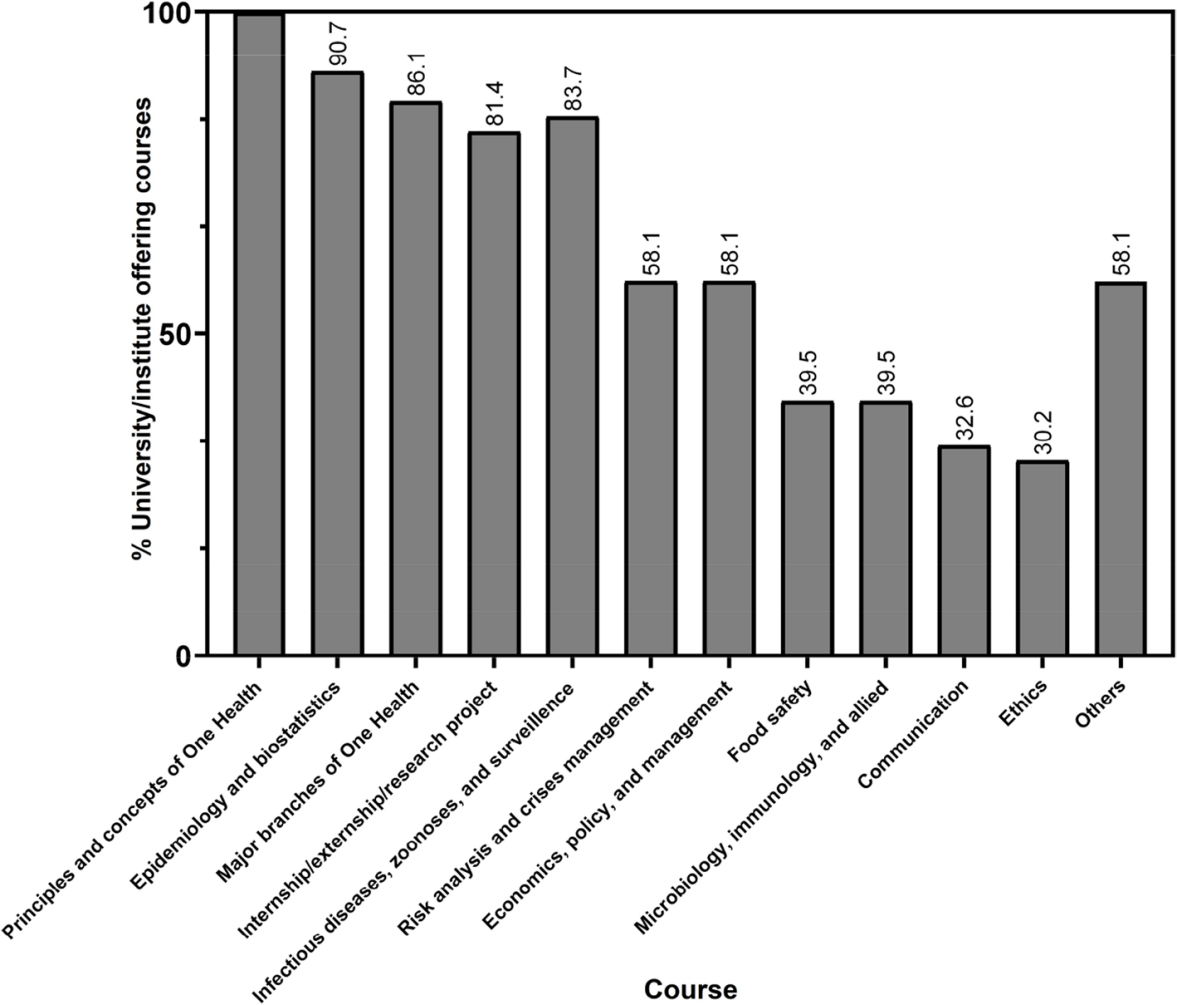
Percentages of curricula contents offered across the One Health (OH) programmes analysed. These curricula contents clustered into 12 different groups and each was assigned a representative sub-heading. Considerable overlap exists between some of the contents of each sub-heading

By default, all courses assessed in this study offered modules on the principles and concepts of One Health (as earlier defined). Epidemiology and biostatistics were taught as distinct modules by 39 (90.7%) OH programmes, and at least two of the three major branches of One Health were taught in 37 programmes (86.1%). However, when taken apart singly, each of the constituting major branches of OH (as earlier defined) was offered in less than 86.1% of the programmes analysed. Thirty-five programmes (81.4%) offered demarcated infectious diseases, zoonoses, and surveillance modules, and mandated that their students undertake a form of internship/externship/research project, and only 25 (58.1%) had distinct risk analysis and crisis management modules. Economics, policy, and management-related modules were included in 25 programmes (58.1%), while in 17 different programmes (39.5%), food safety-related modules, microbiology, immunology, and allied themes were offered. The least modules commonly offered in all the programmes analysed in this study were communications (*n* = 14, 32.6%) and ethics (*n* = 13, 30.2%). Finally, 36 (83.7%) programmes included in this study offered at least one unique module common to less than 10% of the programmes (Fig. 3). Some of these unique contents include (but are not limited to), artificial intelligence, genomics, biosafety and biosecurity, translational medicine, design and architecture, and other basic science courses.

Almost all (*n* = 36, 83.7%) of the OH programmes considered were tailored towards infectious diseases and population/global health, and about 11.6% (*n* = 5) were designed from the environmental health and conservation medicine perspectives. In addition, at least 48.0% (*n* = 21) of programmes analysed in this study, were based in, or organised in conjunction with veterinary institutions compared to at least 23.0% (*n* = 10) in medical institutions. Interestingly, two OH programmes from non-traditional OH allied fields were also identified. The first was designed for architecture and design-related professionals, while the other was for artificial intelligence and data analytics professionals (Supplementary table 1).

## Discussion

As earlier indicated, courses that (1) clearly state ‘One Health’ in their title, or in the degree title to be awarded upon completion, and (2) that are delivered as either a master or a joint bachelor and master programme, in addition, to the exceptions stated in the methodology were included in this study. Based on these inclusion criteria, it was inferred that by default, all the 43 OH programs that met these criteria taught modules on the principles and concept of OH. Modules with themes on infectious diseases and public health constituted the next highest component of the curricula. These modules clustered under the three sub-headings epidemiology and biostatistics; major branches of OH (ecosystem, human and animal health); infectious diseases, zoonoses and surveillance, are taught in at least 81.4% (*n* = 35) of programmes. This observed trend in infectious diseases and zoonoses-centred OH training has also been previously indicated in numerous other studies [1, 10, 16–18].

Historically, the renewed interest in the OH concept was primarily in response to the increased emergence of, and global threats posed by various zoonotic diseases like the H5N1 avian influenza in 1999, severe acute respiratory syndrome (SARS) in 2012, and in more recent times, the Ebola, COVID-19 and Mpox virus diseases [19–21]. However, considering other global threats of OH significance, including environmental deterioration, shortages of food and water, etc.,this imbalanced representation is now considered reductionistic and therefore calls for expanding the focus of OH activities [1, 22]. To this end, the case has been made for expanding the scope of the OH approach (and by extension, the structure of OH education and training) towards the emerging concept of ‘OH systems’ [10]. As Ferrinho and Fronteira [23] described, the traditional OH concept differs from this more recent concept of ‘OH systems’ in that the latter considers a wider scope of sectors, agencies, and actors compared to the former. While such development is optimistic and welcomed, there is scarce literature on the critical analysis of OH systems, and the potential that this emerging sub-field holds is yet to be extensively demonstrated. This, therefore, calls for accompanying concrete data and assessment frameworks to objectively demonstrate the added value of this, and any such systemic innovation [4, 24]. For instance, the recently launched ‘Community of Practice on the Return on Investment for One Health’ (website) [25], supported by the quadripartite group, signifies efforts in this direction.

From the details gathered on the programme websites, there were no indications for the exclusion of any form of research project from eight programmes where this module was absent, and it was not apparent if a variation of the module was incorporated into other modules in the respective curricula. There was also no obvious link between this exclusion and the mode of programme delivery – either online, onsite or hybrid (Supplementary table 1). Furthermore, it was also observed that distinct modules related to risk analysis and crises management were delivered in at least 58.1% (*n* = 25) programmes. However, this observation might not present an objective assessment. ‘Risk and crises’ as a theme is integral to various health sectors. For instance, infectious diseases, epidemiology, and health modules can hardly be taught without mentioning risk analysis and crises management. In essence, while taught modules were used as a proximate in this analysis of curricula contents, this does not infer that the remaining 41.9% (*n* = 18) of the programmes outrightly excluded risk analysis and crises management modules from their curricula.

Seventeen programmes (39.5%) offered modules that clustered under the two sub-headings ‘food safety’ and ‘microbiology, immunology and allied’. Within these sub-headings, a potential overlap of module contents that was alluded to in the preceding paragraph exists. For example, courses that make up the ‘food safety’ sub-heading range from food security, and nutrition, to foodborne zoonoses (Supplementary table 1), while courses that constitute contents of the ‘microbiology, immunology and allied’ sub-heading are rather self-explanatory. In both cases, it can be implied that the outstanding 69.5% (*n* = 26) programmes could have offered some of these constituting courses under a differing sub-heading, for instance, the ‘infectious diseases, zoonoses and surveillance’ sub-heading. This potential overlap also extends to both the ‘communication’ and ‘ethics’ sub-headings. Nevertheless, it can also be inferred that programmes that offer distinct communication and ethics modules would deliver a more robust teaching in comparison to programmes where these subjects or themes are embedded into other bigger modules, like epidemiology.

Five out of twelve sub-headings are common to more than half (*n* = 35) of the total 43 programmes analysed, while another six sub-headings are common to at least 12 and at most 25 programmes. Additionally, 83.7% (*n* = 36) of analysed programmes had at least one module, and at most 12 modules unique to individual programmes which do not fit into any of the twelve sub-headings. This body of data indicates that while there is considerable disparity between all the 43 programmes analysed, there still exists a significant uniformity. An additional highlight from the data generated is that the majority of OH programmes are often designed around the strengths of the organising academic institutions, which was also previously noted by Sidikou et al., [26]. Based on this current study, it was observed that each OH programme gives a bird’s eye view of the OH concept, but despite this, each still emphasises particular subsets of OH. For example, Master in ‘Artificial Intelligence’ for One Health, Master of Science in ‘Infectious Disease and One Health’, and Master in ‘Health and Environment’ (One Health track) approached OH teaching from an artificial intelligence, infectious diseases, and environment point of view respectively. As implied in the One Health Joint Plan of Action [14], the OH workforce does not comprise of esoteric professionals. Rather, it encompasses individuals from diverse backgrounds with skills to effectively communicate, collaborate, build capacity, and coordinate transdisciplinary OH activities. This lends credibility to OH programmes that provide a comprehensive overview of the OH approach while emphasising specific subsets of OH. This also implies that, in the long run, promoting greater diversity in OH programs should be encouraged, provided sufficient uniformity in the modules facilitates the desired cross-talks. This approach, for instance, is gradually becoming commonplace in medical programmes, as reported by Rabinowitz et al., [27], Wilkes et al., [28], and Bayisenge et al., [29]. From this study, it was also observed that inter-institutional collaboration is another alternative approach that has been adopted by a few OH programmes. For instance, the International Joint Master's Degree in Infectious Diseases and One Health, in a true OH approach, is organised and delivered by the faculties of pharmacy, veterinary medicine and a medical school (across three different universities) (Supplementary table 1). A similar approach embraced by the master’s degree in the ‘Zoonoses and One Health' programme is the participation of external specialists from various OH-related fields in delivering the course. This collaborative approach amongst various subsets of OH will go a long way in promoting OH systems, but it should be equally noted that the cost implications involved in such arrangements could be a major bottleneck. A similar difficulty is centred around the potential conflict between the unification of OH systems on a global scale, and the curriculum regulation at the country level to meet national needs. This is aptly reflected in the inability to harmonize global standards for medical education despite the World Federation for Medical Education and the World Health Organization’s best efforts [30].

A large proportion of structured OH programmes subset that met the inclusion criteria of this study were organised in North America and Europe. It was observed that the details and contents of these programmes were readily available and adequately presented on dedicated programme websites. In contrast, few OH programmes from other regions of the world were observed, and the majority of the OH programmes excluded from this review because little or no details were organised in the global south. This further highlights an important deficiency in terms of visibility and accessibility of information about these OH programmes and calls for corresponding improvement by concerned administrators.

Similarly to the OH programmes, literature on OH education and review of teaching contents are majorly available for programmes organised in the global north [9, 11, 26, 31, 32] with only a few studies like Muma et al., [33], Amuguni et al., [8] focusing on similar programmes from Africa, and even lesser from Asia [34]. This observation is in part reflective of the more general issue which has plagued the field of public health for a long time being the unequal distribution of human and capital resources to initiate and sustain some of these programmes in developing countries [23]. Over the years, efforts to bridge this resource gap in terms of OH education have been initiated, especially through international collaborations between the global north and south [8, 34–36] which was also confirmed through data generated from this study. For instance, out of the five OH programmes from Africa that met the inclusion criteria in this study, four were established in conjunction with international collaborators (Supplementary table 1). The One Health analytical epidemiology course at the University of Zambia was initiated together with the London School of Hygiene and Tropical Medicine and the Royal Veterinary College [33], the Master of Science in One Health Molecular Biology at the Sokoine University of Agriculture (within the Africa One Health University Network (AFROHUN)) was designed in conjunction with United States Agency for International Development (USAID) and other international partners (website) [37], Master in Global One Health jointly organised by the Department of Veterinary Tropical Diseases, University of Pretoria, South Africa and the Department of Biomedical Sciences, Institute of Tropical Medicine, Belgium (website) [38], while the Master of Science in One Health (Massive Open Online Course) at The Hassan II Institute of Agriculture and Veterinary Medicine was organised with the support of the European Union (website) [39].

While such collaborations as described above are commendable, some of them are usually time-bound and limited to the period of available funding. Taking collaboration in Asia for example, McKenzie et al., [34] indicated that OH master programmes that were organised in various universities in Afghanistan, Bangladesh, Bhutan, India, Nepal, Pakistan, and Sri Lanka in collaboration with Massey University (and other funding partners) only ran for a period of one to three years. As of this writing, most of these programs are presently run as Master in Public Health programmes, and the extent to which they incorporate the OH concept in their curricula is presently unknown.

Whether OH will evolve into a mainstream field or will remain an approach adopted by already existing health systems has been an interesting subject of speculation [32]. From this study, both models have been used in OH training, i.e. standalone OH programs, and master in public health programs with an OH track. This flexibility has also been described in the literature. A typical case is that of the AFROHUN, where different universities incorporated OH training in diverse ways relative to their individual needs [8]. Since organised OH trainings are still relatively new, it remains to be determined which of these models would have frequent utility in OH education in the long run. However, from the deliverables in the One Health Joint Plan of Action [14], it is evident that a combination of structured degree courses and field training programs will be implemented to strengthen the global OH workforce. It would also be intriguing to observe the extent of influence that various training paths would have on shaping distinct performers in the OH workforce.

Another rare but important development observed in the data collection step of this study was the merger or discontinuation of some OH programmes. For instance, in Reid et al., [31], a list of OH training programs in Australia and New Zealand was provided, but at the time of this writing, details about one specific OH master program on the list could not be fetched (and thus excluded from this study), and it remains unclear whether this programme was merged with another or discontinued. Similarly, the Master in Health and Environment (One Health track) offered at Utrecht University was previously a standalone OH master course (Supplementary table 1). A recent paper by Pinto et al., [40] indicated that OH now forms a core competency in veterinary epidemiology training programs. All these observations further open dialogues about the boundaries of the OH master programmes relative to the already existing health systems.

Lastly, with respect to the contribution of OH programmes to the global OH workforce, data on the hybrid structure of the course delivery and English as the predominant language of instruction indicates the suitability of these programmes to international and multidisciplinary participants. With the disproportionate representation of epidemiology and infectious disease themes in the OH programmes included in this study, it can be cautiously inferred that the career trajectory of graduates of this programme is tilted towards public health, infectious disease, and allied fields, but this can only be objectively proven through a long term follow-up study of OH programme graduates.

This study presents a few limitations. Firstly, the chosen methodology posed a challenge in analysing the incorporation of OH core competencies in OH programs and in determining the extent of this integration. The quantitative or qualitative assessment of competencies like management, leadership, collaboration (as defined in [7], harnessing uncertainty, or system understanding (as defined in [10] based solely on curricula contents proved inadequate. Alternatively, an in-depth pedagogical approach, studying the methods of course delivery and various class exercises (like presentations, group works, gamification, class projects, simulation exercises etc.) would be more suited. This underscores the need for additional studies of OH programmes using a pedagogical approach, similar to Amuguni et al. [35] and Sidikou et al. [26], to determine how OH core competencies are incorporated. Results from these studies could serve as guides for subsequent OHHLEP recommendations.

Additional limitations of this study include the existence of numerous structured OH training activities in higher academic institutions beyond the scope of the inclusion criteria considered, the exclusion of semi-formal OH trainings that falls outside the traditional academic teaching environment, and the overlapping of some modules in the curricula across more than one subheading. Despite these limitations, efforts were made to minimize any confounding effects they might have on data interpretation.

## Conclusions

In conclusion, this study has provided an objective analysis of OH academic programmes, and with this, has bridged a critical information gap that has existed for long in the OH field. Through the generated data, it was established that there exists a disproportionate representation of epidemiology and infectious disease themes in the OH programmes that were included in this study. The data also indicated that the majority of available OH programmes globally are concentrated in developed countries and that through a growing culture of collaboration, more OH trainings are beginning to crop out in other regions of the world. In terms of curricula contents and other parameters that were assessed in this study, it was observed that the diverse OH programs have a significant amount of uniformity, but due to methodological limitations, it was impossible to objectively determine if and how OH core competencies were integrated into these programs. Finally, it was recommended that more pedagogical studies, assessing the alignment of module contents with the OH core competencies be performed.

### Supplementary Information

**Supplementary Material 1.**

## Data Availability

All data generated or analysed during this study are included in this published article and its supplementary information files.
